# Affordable ART in Kenya: The only hope for involuntary childlessness

**Published:** 2016-06-27

**Authors:** SW Ndegwa

**Affiliations:** Footsteps Fertility Foundation, P.O. Box 52848-00100 Nairobi, Kenya.

**Keywords:** Accessible, affordable, infertility, IVF, Kenia, trained personnel

## Abstract

The reported overall subfertility rate in Kenia is 26.1% with 50% attributed to tubal factors and 15% due to male factors. This is probably an underestimation taking into consideration that due to the stigma and myths of the disease, many couples are seeking alternative care from religious sects, witchdoctors, herbalists. Because the costs associated with IVF in private centres are only affordable for the happy few, the only true hope for most Kenyans struggling with unintended childlessness lies in the introduction of affordable ART services. The major challenge is to reduce the costs of ovarian stimulation medication and the equipment set-up cost of the ART laboratory. An important foreseeable barrier to low-cost ART is adequately trained personnel.

## Introduction

Infertility is one of the key components of Reproductive Health, yet it remains an entirely neglected problem in Kenya. Data on infertility in Kenya and much of Sub Saharan Africa is scanty though the few existing studies show that the region is grossly affected by the problem of infertility. In his study on global access to infertility care in developing countries, Ombelet (2012) states that more than 180 million couples in developing countries suffer from primary or secondary infertility In Kenya, the overall rate of subfertility was 26.1% among gynaecology consultations, with 50% attributed to tubal factors and 15% due to male factors (Murage A et al., 2011). This is still grossly underestimated taking into consideration that due to the stigma and myths of the disease, the couples seeking alternative care from religious sects, witchdoctors, herbalists and those whose quest for a child ends at the diagnosis of infertility.

Kenya has a population of 38.6 Million people with a total fertility rate of 4.6% in 2009 (KDHS, 2010). In a country whose main priority is to reduce the growing population size through government and donor funded reproductive health programmes in support of contraception and safe abortions, it is not surprising that Kenya has no provision for public fertility care. It is simply not a priority. The debate surrounding infertility care in developing countries has been that, for countries burdened by high growth rates and poverty, focus should be on contraception and other population control programmes. However, there is no evidence from developed countries where infertility care is mainstreamed that links infertility care to increased population growth. In fact, evidence suggest the opposite since countries like Egypt have been highly successful in controlling population growth by integrating contraception services with infertility care for childless couples (Serour at al., 2008).

Currently there are six private fertility centres in Kenya, performing a total of approximately 900 IVF/ICSI cycles per year. The global need for IVF services has been estimated to be at least 1,500 cycles per million (Ombelet et al., 2008). We have a huge unmet need. One of the reasons to poor accessibility is the high cost of IVF/ICSI, with the lowest cost per cycle being $4500 per treatment. In a country where the poverty levels remain at 42%, as estimated by a 2013 World Bank report, the cost of ART is out of reach for many, making the dream of a child for the infertile couple painfully impossible.

Ironically, the less educated, rural­-poor Kenyans and those living in urban informal settlements face the most devastating consequences of infertility. In the African context and still largely in Kenya the significance of a woman is solely defined through motherhood. Childless couples in Sub­-Saharan Africa face the most severe negative psychosocial consequences and childless women are frequently stigmatized, isolated, ostracized, disinherited and neglected by the entire family and even the local community (Daar and Merali, 2002). The family, including siblings, parents and in­laws are deeply disappointed for the loss of continuity of their family and contribution to their community. This amplifies the guilt and shame felt by the infertile individual. This often results in polygamy and severe physical and psychological violence mitigated towards the woman, driving some infertile women to suicide (Anonymous, 2006). Additionally, emerging evidence suggests that infertile women, especially in Sub-­Saharan Africa, are more likely than fertile women to be exposed to the HIV as a result of extramarital attempts to conceive (Bentley et al., 2000). Men also suffer stigma from their infertility. Male infertility (estimated to be the cause of infertility in up to 50% of cases) is often deeply hidden in most societies because it is among the most stigmatizing of all male health conditions (Inhorn, 2000). Unintended childlessness is associated with overwhelming pain and grief that is often underestimated.

Fertility care is a basic right for every human being. At the United Nations International Conference on Population and Development in Cairo, Reproductive Health was defined as people having the right to decide when to have children, the number and the means to do so. This reproductive autonomy has been the main argument in favour of the provision of infertility treatment in developed countries. If citizens of developed countries enjoy this right, why would citizens of developing countries not have the right to have at least one child, especially if infertility care is made more affordable for a much larger part of the population (Ombelet, 2012)? If overpopulation was the threat, would accessibility to infertility treatment in developing countries account for more than 1% of all deliveries (Ombelet, 2011)? Given that inclusivity principles are universally encouraged in building a cohesive society, does ignoring infertile individuals amount to social justice and equity? Finally, does the presence of more pressing health needs to be coupled with the challenge of limited resources warrant total disregard to infertility treatment?

The only true hope for most Kenyans struggling with unintended childlessness lies in the introduction of affordable ART services. The challenge is to reduce the costs of medication and the equipment set up cost of the ART laboratory. Modified natural and mild stimulation IVF protocols have not yet been implemented in Kenya. This approach may be the only feasible solution to the infertile couple especially since the burden of infertility is largely attributed to bilateral tubal occlusion, which is bypassed by this technique. ART at an affordable cost would allow couples to seek fertility care early before age related ovarian factors affect favourable outcomes. Modified natural and mild stimulation IVF with single embryo transfer will also address some of the costly complications of conventional IVF such as multiple pregnancy, ovarian hyper­ stimulation syndrome and premature new­borns. The use of equipment that can be purchased at lower prices, and possibly simpler, less costly laboratory and clinical infrastructure also serve to reduce the overall cost.

The only foreseeable barrier to low cost ART is adequately trained personnel. Currently, in Kenya there are less than ten well­trained IVF specialists and no adequate trained embryologist. Most centres hire an expatriate embryologist who charges a very high rate per patient per cycle, forcing these centres to operate in ART batch cycles to reduce on costs. We must train interested Kenyans if we truly want to implement and sustain affordable ART in the near future.

The extensive attention ART is receiving around Kenyan media, as we are currently debating on the legislation of ART, will hopefully serve to sensitize the public on the need for affordable fertility care. Egypt has made remarkable progress in integrating ART into the public sector through government support and subsidies that ensure infertile couples get a chance to become parents. Low cost fertility services in Kenya may make access feasible in at least some settings and drive the momentum for government involvement. Affordable ART in Kenya will give the much needed hope to the now hopeless couples struggling with involuntary childlessness.

## References

[1] (2006). Cheap IVF needed. (editorial). Nature.

[2] Bentley GR, Mascie­Taylor N (2000). Infertility in the Modern World: Present and Future Prospects.

[3] Daar AS, Merali Z (2002). Infertility and social suffering: the case of ART in developing countries. Vayena E, Rowe PJ, Griffin PD (9eds). Current Practices and Controversies in Assisted Reproduction.

[4] Inhorn MC (2004). Middle Eastern masculinities in the age of new reproductive technologies: male infertility and stigma in Egypt and Lebanon.. Med Anthr Quart.

[5] http://www.worldbank.org/content/dam/Worldbank/document/Africa/Kenya/kenya-economic-update-june-2013.pdf.

[6] Kenya National Bureau of Statistics (KNBS) and ICF Macro 2010 Kenya Demographic and Health Survey 2008-09. Calverton, Maryland: KNBS and ICF Macro http://www.unfpa.org/sowmy/resources/doc/library/R313_KNBS_2010_Kenya_DHS_2009_final_report.pdf

[7] Murage A, Muteshi MC, Githae F (2011). Fertil Steril. Assisted reproduction services provision in a developing country: time to act.

[8] Serour GI (2008). Medical and socio­cultural aspects of infertility in the Middle East. ESHRE Monograph.

[9] Ombelet W, Cooke I, Dyer S (2008). Infertility and the provision of infertility medical services in developing countries.. Hum Reprod Update.

[10] Ombelet W (2011). Global access to infertility care in developing countries: a case of human rights, equity and social justice.. Facts Views Vis Obgyn.

